# Postpartum Medicaid coverage and outpatient care utilization among low-income birthing individuals in Oregon: impact of Medicaid expansion

**DOI:** 10.3389/fpubh.2023.1025399

**Published:** 2023-07-04

**Authors:** Satyasandipani Pradhan, S. Marie Harvey, Linh N. Bui, Jangho Yoon

**Affiliations:** ^1^School of Social and Behavioral Health Sciences, College of Public Health and Human Sciences, Oregon State University, Corvallis, OR, United States; ^2^Public Health Program, School of Natural Sciences, Mathematics, and Engineering, California State University, Bakersfield, CA, United States; ^3^Department of Preventive Medicine and Biostatistics, F. Edward Hebert School of Medicine, The Uniformed Services University of the Health Sciences, Bethesda, MD, United States

**Keywords:** medicaid expansion, medicaid, coverage, postpartum, maternal health, affordable care act, outpatient, access and use

## Abstract

**Objective:**

This study examined the effect of Medicaid expansion in Oregon on duration of Medicaid enrollment and outpatient care utilization for low-income individuals during the postpartum period.

**Methods:**

We linked Oregon birth certificates, Medicaid enrollment files, and claims to identify postpartum individuals (*N* = 73,669) who gave birth between 2011 and 2015. We created one pre-Medicaid expansion (2011–2012) and two post-expansion (2014–2015) cohorts (i.e., previously covered and newly covered by Medicaid). We used ordinary least squares and negative binomial regression models to examine changes in postpartum coverage duration and number of outpatient visits within a year of delivery for the post-expansion cohorts compared to the pre-expansion cohort. We examined monthly and overall changes in outpatient utilization during 0–2 months, 3–6 months, and 7–12 months after delivery.

**Results:**

Postpartum coverage duration increased by 3.14 months and 2.78 months for the post-Medicaid expansion previously enrolled and newly enrolled cohorts (*p* < 0.001), respectively. Overall outpatient care utilization increased by 0.06, 0.19, and 0.34 visits per person for the previously covered cohort and 0.12, 0.13, and 0.26 visits per person for newly covered cohort during 0–2 months, 3–6 months, and 7–12 months, respectively. Monthly change in utilization increased by 0.006 (0–2 months) and 0.004 (3–6 months) visits per person for post-Medicaid previously enrolled cohort and decreased by 0.003 (0–2 months) and 0.02 (7–12 months) visits per person among newly enrolled cohort.

**Conclusion:**

Medicaid expansion increased insurance coverage duration and outpatient care utilization during postpartum period in Oregon, potentially contributing to reductions in pregnancy-related mortality and morbidities among birthing individuals.

## Introduction

The United States has one of the highest rates of deaths among birthing individuals due to complications from pregnancy and childbirth among developed nations (1). The postpartum period from delivery through 6 months after delivery (2–4) is a critical time in the lives of birthing individuals and their newborns. Appropriate healthcare during this period can reduce mortality and morbidities such as urinary incontinence, depression, diabetes, and cardiac diseases among others (5–7) in the birthing individuals. Importantly, morbidity and mortality occur at higher rates among low-income birthing individuals in minority racial/ethnic groups, who also disproportionately receive maternity care through the Medicaid program.

The guidelines for postpartum care from the American College of Obstetrics and Gynecologists (2018) recommended comprehensive postpartum visits through 12 weeks postpartum (2). However, Medicaid only provides postpartum coverage from conception to 60 days after childbirth (2) for those low-income postpartum individuals who obtained pregnancy-related Medicaid coverage. After 60 days, these individuals can remain enrolled in Medicaid only if they are eligible for parental Medicaid or Child Health Insurance Program (CHIP) coverage based on income levels. This gap in coverage can render some birthing individuals without health insurance, affecting their access to postpartum care (8). Lack of health insurance may also contribute to these individuals and their families suffering from negative financial impact due to high out-of-pocket expenses related to healthcare needs (9).

Birthing individuals who do not receive appropriate postpartum care are at higher risk for undiagnosed complications of pregnancy, delayed initiation of contraception, and unaddressed infant care issues (10). In spite of the importance of postpartum care, only 40% of birthing individuals enrolled in Medicaid received postpartum care (2). This low utilization of care may be reflective of general barriers to healthcare access and utilization, as documented in previous literature (11–13).

In an effort to reduce health disparities and gaps in coverage for low-income people, the Affordable Care Act (ACA) provides the opportunity for states to expand Medicaid eligibility to adults with incomes up to 138% of the federal poverty level (FPL) through an optional Medicaid expansion (14). As of March 2021, 39 states and the District of Columbia had expanded Medicaid eligibility (15). Estimates indicated that by 2016 more than 20 million adults were enrolled through the ACA Medicaid expansion; 9.5 millions of these enrollees were individuals who could potentially give birth (16). Increased enrollment in Medicaid potentially improves postpartum insurance coverage and access to care.

Despite the importance of postpartum care and the far reaching social and individual impacts of such care on birthing individuals and their families, the effect of Medicaid expansion on postpartum coverage and use of postpartum care remains an understudied area. Although the ACA is associated with a decrease of up to 21% in the uninsurance rate for the general population (17), Marchi et al. (18) reported almost a 10% reduction in uninsurance rates during the postpartum period following the implementation of Medicaid expansion. In recent studies that examined the impact of Medicaid expansion on continuous enrollment in postpartum care, researchers found increased probabilities of having Medicaid coverage and postpartum care utilization (19–22). In addition, the ACA Medicaid expansion in Colorado and Arkansas were associated with greater use of outpatient care in the postpartum period compared to Utah, a non-expansion state (7, 21). Medicaid expansion was also found to have a significant association with a reduction in the mortality ratio among individuals who gave birth (23).

In 2014, under the ACA, Oregon expanded Medicaid eligibility to all individuals with incomes between 100 and 138% of the FPL. During the same period, Oregon also raised the income limit for Medicaid eligibility for pregnancy-related eligibility from 185 to 190% FPL. These extensions potentially increased postpartum duration of coverage and outpatient healthcare utilization for women who were previously enrolled and newly enrolled in Medicaid. Previous research suggested that it takes time for those newly enrolled in Medicaid to utilize healthcare services at the same rate as those who were previously enrolled (24).

By 2018 Oregon had enrolled 490,700 newly eligible beneficiaries under the expansion criteria (24). The aims of this study were, therefore, to (1) evaluate the impact of Medicaid expansion on the duration of Medicaid enrollment during the postpartum period, and (2) examine the effect of Medicaid expansion on utilization of outpatient care, among low-income birthing individuals in Oregon.

## Methods

### Study population and study cohorts

The study population included all birthing individuals aged 18–44 in Oregon who had at least one delivery funded by Medicaid between 2011 and 2015. We divided our sample into three delivery cohorts, one pre-Medicaid expansion and two post-Medicaid expansion cohorts. Pre-Medicaid expansion deliveries occurred during 2011–2012 and were not impacted by Medicaid expansion. We excluded deliveries in 2013 because a portion of the 12-months postpartum period occurred in 2014 when Medicaid expansion was implemented. Post-Medicaid expansion cohorts included deliveries in 2014 and 2015. Deliveries during the 2014–2015 period to individuals who ever had Medicaid coverage prior to 2014 were categorized as the post-Medicaid expansion previously enrolled cohort. We assumed that individuals who had never been enrolled in Medicaid before 2014 were new enrollees who gained coverage due to Medicaid expansion. Therefore, deliveries during the 2014–2015 period by these birthing individuals were defined as the post-Medicaid expansion newly enrolled cohort (Appendix Figure 1).

### Data sources

We linked person-level datasets acquired from different sections or programs within the Oregon Health Authority (OHA) and included Oregon Medicaid eligibility and claims, birth certificates, death certificates and hospital discharge data of individuals who gave birth during the study period. The linkage was completed by the Integrated Client Service data warehouse maintained by the Oregon Health Authority (OHA) and the Oregon Department of Human Services (ODHS). The linked dataset captures all Medicaid funded births in the state of Oregon. Medicaid enrollment files were used to calculate the duration of postpartum coverage and to track historical coverage status for the cohort creation. Claims data were used to identify utilization of outpatient care services by birthing individuals after delivery. Individuals’ socio-demographic and birth characteristics were obtained from Medicaid enrollment files and birth certificates. The study was approved by institutional review boards at the [blinded for review].

### Measures

#### Dependent variables

##### Medicaid postpartum coverage duration

A count variable was created that indicated the total number of months of Medicaid coverage from delivery date to the last day of the 12-months follow-up period or the last month of Medicaid enrollment. An individual was counted as having coverage for the entire month if they were enrolled for at least 1 day in the month.

##### Utilization of outpatient care

Centers for Medicare and Medicaid Services define outpatient care as medical or surgical care received from a clinic or hospital but not admitted as an inpatient. Outpatient care may include emergency department services, observation services, outpatient surgery, lab tests, or X-rays (25). We defined outpatient care as all medical services with the exception of inpatient and emergency care. We examined total number of outpatient visits that occurred in three postpartum periods: 0–2 months, 3–6 months, and 7–12 months and used Current Procedural Terminology (CPT) codes (see Appendix) in Medicaid claims file to identify these visits.

### Primary independent variables and covariates

We included an indicator for post-Medicaid expansion delivery that took a value of 1 if the delivery occurred during 2014 or 2015. The coefficient of this variable estimated the average difference in each outcome between the pre-Medicaid expansion and the post-Medicaid expansion cohorts. We created binary indicators for the three delivery cohorts: pre-Medicaid expansion, post-Medicaid expansion previously enrolled, and post-Medicaid expansion newly enrolled. We also created a time trend variable to investigate how the impact of Medicaid expansion on the outcomes changed over the post-Medicaid expansion timeline. This variable took the value of 0 for all deliveries during the pre-Medicaid expansion period and January 2014 when Medicaid expansion started, and increased by 1 for each month thereafter till December of 2015. We also included the interaction terms of the time trend variable and indicators of post-Medicaid expansion cohorts to estimate the monthly change of the impact of Medicaid expansion on the outcomes during the Medicaid expansion period, compared to the pre-Medicaid expansion period.

Our statistical models examining the impact of Medicaid expansion on the outcomes controlled for individual level characteristics were collected from birth certificate data including age, race/ethnicity, educational status, marital status, rurality, type of delivery, parity, number of prenatal care visits, and morbidities among infants and birthing individuals. Birthing individual’s age was a continuous variable that denoted the age of the individual during delivery and ranged from 18 to 44 years. Binary variables for the individuals’ race-ethnicity included Non-Hispanic (NH) white, NH Black, NH American Indian and Alaskan Native (AIAN), NH Asian, Native Hawaiian and Pacific Islander, and Hispanic ethnicity. The educational status of the individual during delivery was denoted by binary variables and included less than high school education, high school and some college education, bachelor’s degree, and graduate degree. Type of delivery was denoted by binary variables and included spontaneous delivery, assisted vaginal delivery, and cesarean delivery. Binary variables for parity included zero, one, two, and three or more deliveries. A count variable for total prenatal visits for the delivery was also used and ranged from 0 to 55 visits.

Morbidities among birthing individuals included diabetes, hypertension during pregnancy, and eclampsia. Infant morbidities included premature birth, congenital infections, and low birth weight. A three-level rurality variable which included urban, large rural town, small and isolated rural towns was created from ZIP code of residence using the Rural Urban Commuting Area Categorization B (RUCA) criteria (26). Cases with missing data were excluded from our analysis. However, because the proportion of missing data was relatively low, this exclusion did not significantly impact the results of our study.

### Analytic approach

We estimated descriptive statistics for dependent and independent variables by cohorts and compared characteristics between pre-Medicaid expansion group and each of post-Medicaid expansion groups using unadjusted bivariable regression models. We used multivariate ordinary least squares regression to estimate the impact of Medicaid expansion on the duration of postpartum coverage during the first 12 months following delivery.

We used negative binomial regression models to estimate the impact of Medicaid expansion on service utilization in the post-Medicaid expansion period compared to the pre-Medicaid period, adjusting for full list of covariates. We estimated two sets of models, including models comparing the pre-Medicaid expansion cohort and the post-Medicaid expansion previously enrolled cohort, and models comparing the pre-Medicaid expansion cohort and the post-Medicaid expansion newly enrolled cohort. Because coefficients from nonlinear models could be misleading and do not capture magnitude, we computed the average marginal effect (via a finite difference method) that measured an average change in outpatient utilization associated with Medicaid expansion. Because the mathematical structure of the standard error of the average marginal effect is unknown, we used bootstrapped standard errors (300 repetitions) for statistical significance tests.

Model robustness was confirmed after conducting sensitivity analysis with zero inflated negative binomial (ZINB) models. While the results were very similar, the negative binomial models exhibited better Akaike information criterion, Bayesian information criterion, and log-likelihood values when compared with ZINB models. We also conducted sensitivity analysis for the impact of Medicaid expansion on postpartum outpatient care utilization by excluding individuals who had multiple births during the study period.

## Results

### Sample description

The sample included 73,669 deliveries, 35,513 deliveries (48.21%) in 2011–2012 prior to Medicaid expansion and 38,155 deliveries (51.79%) in the post-Medicaid expansion period 2014–2015 (Table 1). Among post-Medicaid expansion deliveries, 29,514 (40.06%) were post-Medicaid expansion previously enrolled and 8,642 (11.73%) deliveries were post-Medicaid expansion newly enrolled.

**Table 1:** Characteristics of Medicaid-enrolled birthing individuals in Oregon by expansion cohort, 2011–2015.

Characteristic	Pre – Medicaid expansion cohort		Post-Medicaid expansion cohorts (39,220)				Total	
			Previously enrolled cohort^1^		Newly enrolled cohort^2^			
	35,513 (48.21%)		29,514 (40.06%)		8,642 (11.73%)		73,669 (100%)	
	Frequency	%	Frequency	%	Frequency	%	Frequency	%
Mean Age (years)	26.55 (SD = 5.59)		27.00^**^ (SD = 5.55)		27.53^***^ (SD = 5.56)		26.84 (SD = 5.58)	
Race/Ethnicity								
NH White	20,763	58.47	17,971^***^	60.89	5,389^***^	61.94	44,123	59.89
NH Black	1,080	3.04	931	3.15	199	2.30	2,210	3.00
NH AIAN	1,010	2.84	732	2.48	435	5.03	2,177	2.96
NH Asian	670	1.89	499	1.69	124	1.43	1,293	1.76
NH PI	450	1.27	267	1.17	128	1.48	924	1.25
NH Other Race	267	0.75	126	0.43	79	0.91	472	0.64
Hispanic	11,185	31.50	8,855	30.00	2,270	26.27	22,310	30.28
Missing	88	0.25	53	0.18	18	0.21	159	0.22
Education								
Less than HS	10,351	26.88	7,934^***^	26.88	1,584^***^	18.33	19,985	26.97
HS and some college	20,993	59.11	18,125	61.41	5,153	59.63	44,271	60.10
Bachelor’s degree	3,599	10.13	2,984	10.11	1,567	18.13	8,150	11.06
Graduate degree	391	1.10	328	1.11	290	3.36	1,009	1.37
Missing	179	0.50	142	0.48	48	0.56	369	0.50
Residence								
Urban	30,332	85.41	23,664^***^	80.18	7,026^***^	81.30	61,022	82.83
Large Rural	3,756	10.58	4,329	14.67	1,202	13.91	9,287	12.61

^***^p < 0.001, ^**^p < 0.01.

*p*-values were obtained from unadjusted bivariable regression models.

NH, Non-Hispanic; AIAN, American Indian Alaskan Native; PI, Pacific Islander.

1 All characteristics were compared between Post Medicaid-expansion Previously enrolled cohort and Pre-Medicaid expansion cohort.

2 All characteristics were compared between Post Medicaid-expansion Newly enrolled cohort and Pre-Medicaid expansion cohort.

*T*-test was used for age and Chi-square test was used for all other variables when comparing cohorts.

The mean age of birthing individuals at the time of delivery was 26.84 years (standard deviation [*SD*] = 5.58). Compared to the pre-Medicaid expansion cohort (mean = 26.55; [*SD*] = 5.59), birthing individuals in both post-Medicaid expansion previously enrolled cohort (mean = 27.00; [*SD*] = 5.55) and post-Medicaid expansion newly enrolled cohort (mean = 27.53; [*SD*] = 5.56) were older (*p* < 0.001). Racial/ethnic composition was similar among the three delivery cohorts. Non-Hispanic (NH) White birthing individuals formed the majority racial and ethnic group, followed by birthing individuals who identified as Hispanic. Less than 10% of the birthing individuals identified as NH Black, NH American Indian and Alaskan Native, NH Pacific Islander and Other NH races. Birthing individuals in post-Medicaid expansion newly enrolled cohort were more likely to have bachelors’ degree and post-graduate degree compared to both the pre-Medicaid expansion and post-Medicaid expansion previously enrolled cohorts (*p* < 0.001). The proportion of birthing individuals living in rural areas was higher for the post-Medicaid expansion previously enrolled (18.99%) and the post-Medicaid expansion newly enrolled cohorts (17.84%), compared to the pre-Medicaid expansion cohort (17.68%) (*p* < 0.001).

### Medicaid expansion and duration of Medicaid enrollment

We visually examined monthly trends in duration of enrollment by cohort and observed an increase for both the post-expansion cohorts with birthing individuals in the post-Medicaid expansion previously enrolled cohort having longer coverage duration by the end of 2016 (Appendix Figure 2). In adjusted analysis, we observed that duration of postpartum coverage increased for the post-Medicaid expansion previously enrolled and the post-Medicaid expansion newly enrolled cohorts (Table 2). Coverage duration for deliveries of the post-Medicaid expansion previously enrolled cohort increased by 3.14 months (*p* < 0.001; 95% CI: 2.92, 3.09) compared to the pre-Medicaid expansion cohort. This change represented a 37.66% increase compared to the baseline mean coverage duration of 8.31 months (Table 3) among pre-Medicaid expansion deliveries. Medicaid expansion was associated with an increase of 2.78 months (*p* < 0.001; 95% CI: 3.05, 3.35) or 33.4% increase in coverage duration for the post-Medicaid expansion newly enrolled deliveries, compared to pre-Medicaid expansion deliveries.

**Table 2:** Impact of Medicaid expansion on duration of enrollment during the 12-month postpartum period for Medicaid-enrolled birthing individuals of Oregon in 2011–2015.

Birth cohorts and covariates	No. of months covered after delivery	
	Change^1^	95% CI
Post-Medicaid expansion previously covered cohort	3.14^***^	2.92–3.09
Post-Medicaid expansion newly covered cohort	2.78^***^	3.05–3.35
Time trend	−0.06^***^	−0.06 – –0.05
Individual’s age	−0.00	−0.00 – 0.00
RUCA (Reference: Urban)		
Large rural	−0.21^***^	−0.30 – –0.15
Small and Isolated rural	−0.23^***^	−0.36 – –0.11
Race/Ethnicity (Reference: White)		
NH Black	0.66^***^	0.52–0.81
NH Asian	0.00	−0.15 – 0.15
NH AIAN	0.20^*^	0.01–0.39
NH PI	−0.37^**^	−0.60 – –0.15
NH Others	−0.11	−0.43 – 0.19
Hispanic	−0.40^***^	−0.46 – –0.33
Individuals’ Education (Reference: Less than Highschool)		
Highschool and some college	−0.44^***^	−0.50 – –0.38
Bachelor’s degree	−0.91^***^	−1.00 – –0.81
Graduate degree	−1.12^***^	−1.34 – –0.91
Marital status (Reference: Unmarried)		
Married	−0.62^***^	−0.67 – –0.57
Mode of delivery (Reference: Spontaneous)		
Assisted vaginal	−0.22^*^	−0.37 – –0.07
Cesarean	0.05	−0.00 – 0.11
Morbidity in the birthing individual (Reference: Absent)		
Present	−0.12	−0.26 – 0.03
Infant morbidity (Reference: Absent)		
Present	0.12^*^	0.04–0.20
Previous pregnancies (Reference: No previous pregnancies)		
1	0.13^***^	0.06–0.19
2	0.36^***^	0.26–0.45
> = 3	0.42^***^	0.30–0.53
Antenatal visits (Reference: Zero antenatal visits)		
> = 1	0.01^*^	0.00–0.02

^***^p < 0.001, ^**^p < 0.01, ^*^p < 0.05.

NH, Non-Hispanic; AIAN, American Indian Alaskan Native; PI, Pacific Islander.

1 Reference group was Pre-Medicaid expansion cohort.

Results from multivariate regression models are adjusted for time trend, individual’s age, race/ethnicity, education, rurality, marital status, type of delivery, presence of morbidity in the birthing individual, infant morbidity, parity, and number of prenatal visits attended.

**Table 3:** Baseline characteristics of Medicaid access and utilization for Medicaid-enrolled birthing individuals in Oregon by expansion cohort, 2011–2015.

Characteristic	Pre – Medicaid expansion cohort		Post-Medicaid expansion cohorts (39,220)				Total	
			Previously enrolled cohort^1^		Newly enrolled cohort^2^			
	35,513 (48.21%)		29,514 (40.06%)		8,642 (11.73%)		73,669 (100%)	
	Frequency	%	Frequency	%	Frequency	%	Frequency	%
Mean duration of coverage (in months)	8.31 (4.17)		10.83 (2.23)^***^		10.39 (2.67)^***^			
Mean frequency of outpatient care visits								
0–2 months	0.95 (SD = 1.36)		1.07^***^		1.06^***^			
3–6 months	0.56 (SD = 1.40)		0.79^***^		0.62^**^			
7–12 months	0.80 (SD = 1.95)		1.12^***^		0.84^***^			

^***^p < 0.001, ^**^p < 0.01.

*p*-values were obtained from unadjusted bivariable regression models.

1 All characteristics were compared between Post Medicaid-expansion Previously enrolled cohort and Pre-Medicaid expansion cohort.

2 All characteristics were compared between Post Medicaid-expansion Newly enrolled cohort and Pre-Medicaid expansion cohort.

*T*-test was used for all variables when comparing cohorts.

### Medicaid expansion and outpatient care utilization

The results of our analyses examining the effect of the Medicaid expansion on number of outpatient care visits by postpartum periods are presented in Table 4. During the 0–2 months postpartum period, on average, the utilization of outpatient care by the post-Medicaid expansion previously enrolled birthing individuals was higher by 0.06 visits per person (*p* < 0.001; 95% CI: 0.03, 0.09) compared to that of pre-Medicaid expansion birthing individuals. The mean frequency of outpatient care visits for the post-Medicaid expansion previously enrolled cohort was, thus, higher at 1.02 visits per person, when compared to the baseline of 0.95 visits per person (Table 3) in the pre-Medicaid expansion cohort. For every month further from the start of Medicaid expansion, the monthly change in average number of visits during 0–2 months postpartum for previously enrolled birthing individuals was higher by 0.006 visits per person (*p* < 0.001; 95% CI: 0.004, 0.009) compared to that of the pre-Medicaid expansion birthing individuals. Overall, the post-Medicaid expansion newly enrolled cohort had more outpatient visits during the 0–2 months postpartum by 0.12 visits per person (*p* < 0.001; 95% CI: 0.06, 0.17) compared to the pre-Medicaid expansion birthing individuals. Compared to the pre-Medicaid expansion period, monthly change in utilization of outpatient care during 0–2 month postpartum for newly enrolled birthing individuals was lower by 0.003 visits per person (*p* < 0.001; 95% CI: −0.007, 0.002).

**Table 4:** Negative binomial regression models on number of outpatient care visits by postpartum periods for Medicaid-enrolled Oregon birthing individuals in 2011–2015.

Postpartum periods	Post-Medicaid expansion previously enrolled cohort		Post-Medicaid expansion newly enrolled cohort	
	Change^1^	95% CI	Change^1^	95% CI
0–2 months				
Average number of visits per person during post-Medicaid expansion period (overall)	0.06^***^	0.03–0.09	0.12^***^	0.06–0.17
Monthly change in average number of visits per person	0.006^***^	0.004–0.009	−0.003^***^	−0.007 – 0.002
3–6 months				
Average number of visits per person during post-Medicaid expansion period (overall)	0.19^***^	0.16–0.22	0.13^***^	0.07–0.19
Monthly change in average number of visits per person	0.004^*^	0.001–0.007	−0.005	−0.01 – 0.00
7–12 months				
Average number of visits per person during post-Medicaid expansion period (overall)	0.34^***^	0.30–0.39	0.26^***^	0.17–0.36
Monthly change in average number of visits per person	−0.002	−0.007 – 0.002	−0.02^***^	−0.03 – –0.01

^***^p < 0.001, ^*^p < 0.05.

1 Reference group was Pre-Medicaid expansion cohort.

Results are marginal effects estimated from negative binomial regression models. Models were adjusted for time trend, individual’s age, race/ethnicity, education, rurality, marital status, type of delivery, presence of morbidity in the birthing individuals, infant morbidity, parity, and number of prenatal visits attended.

The full regression results from the negative binomial regression models are presented in Appendix Tables 5–11.

In the 3–6 months postpartum period, on average, utilization of outpatient care by the post-Medicaid expansion previously enrolled birthing individuals was higher by 0.19 visits per person (*p* < 0.001; 95% CI: 0.16, 0.22) compared to that of pre-Medicaid expansion birthing individuals. Therefore, the average number of outpatient visits among the post-Medicaid expansion previously enrolled cohort was higher at 0.75 visits per person compared to the baseline service utilization of 0.56 visits per person (Table 3) by the pre-Medicaid expansion cohort. For each incremental month during the post-Medicaid expansion period, monthly change in service utilization during 3–6 month postpartum for previously enrolled birthing individuals was higher by 0.004 visits per person (*p* < 0.05; 95% CI: 0.001, 0.007) compared to that of pre-Medicaid expansion birthing individuals. We found that the post-Medicaid expansion newly enrolled cohort had an increase in outpatient visits during this postpartum period by 0.13 visits per person (*p* < 0.001; 95% CI: 0.08, 0.20) compared to that of pre-Medicaid expansion birthing individuals. Although not statistically significant, compared to the pre-Medicaid expansion period, monthly change in utilization of outpatient care during 3–6 month postpartum for newly enrolled birthing individuals was lower by 0.005 visits per person visits (*p* = 0.09; 95% CI: −0.01, 0.00).

Outpatient care utilization within 7–12 months postpartum due to Medicaid expansion increased on average by 0.34 visits per person (*p* < 0.001; 95% CI: 0.30, 0.39) and 0.26 visits per person (*p* < 0.001; 95% CI: 0.18, 0.37) for birthing individuals in post-Medicaid expansion previously enrolled and post-Medicaid expansion newly enrolled cohorts, respectively, compared to that of birthing individuals in pre-Medicaid expansion cohort. For every month since the Medicaid expansion in January 2014 up to the end of 2016, the average utilization of outpatient care within 7–12 months postpartum on average decreased by 0.002 visits per person (*p* = 0.29; 95% CI: −0.006, 0.002) and 0.02 visits per person (*p* < 0.001; 95% CI: −0.03, −0.01) for the post-Medicaid expansion previously enrolled cohort and post-Medicaid expansion newly enrolled cohorts, respectively, compared to that of pre-Medicaid expansion cohort. Sensitivity analysis conducted after excluding individuals with multiple deliveries yielded similar results, establishing the robustness of our results.

## Discussion

Our study is the first to explore the impact of Medicaid expansion on postpartum coverage and outpatient care among Medicaid-enrolled birthing individuals in Oregon. We found that the expansion of Medicaid in Oregon increased both duration of Medicaid coverage and utilization of outpatient care services in the postpartum period for low-income birthing individuals. We also found that birthing individuals who were previously enrolled in Medicaid had a larger increase in Medicaid coverage duration compared to newly enrolled birthing individuals.

After Medicaid expansion, birthing individuals who were previously enrolled in Medicaid experienced a larger increase in use of outpatient care compared to new enrollees during 3–6 months and 7–12 months after delivery. However, during the 0–2 months postpartum period, birthing individuals who were newly enrolled in Medicaid had more outpatient visits than those previously enrolled in the program.

Our findings are consistent with previous research that examined the effect of Medicaid expansion on use of services during the post-partum period (7, 19). These studies examined the impact of expansion for 6 months (7) or 300 days (19) post-delivery. We expanded the time frame to explore outpatient utilization trends over three time periods during the first 12 months post-delivery. This approach allowed us to better understand the trends in use during the postpartum period and the long-term impact of the policy on postpartum care utilization. Tracking access and utilization over 12 months after delivery also aligns with the provision under the American Rescue Plan which extended Medicaid coverage for pregnant people from 2 to 12 months after delivery (27).

Pent up demand could be one of many potential reasons for the increase in utilization in both post-expansion cohorts compared to the pre-expansion birthing individuals birthing individuals (28). Extension of pregnancy related medicaid eligibility from 185 to 190% FPL may also have contributed to some increase in utilization of services during the 0–2 months postpartum period. The largest increase in outpatient care utilization for both post-Medicaid expansion cohorts was during the 7–12 months postpartum period, followed by the increase in utilization in the 3–6 months postpartum. These periods coincide with the time frame when new birthing individuals would have lost coverage prior to Medicaid expansion. This finding could, in part, be due to delays in receiving services that newly enrolled birthing individuals may have experienced. Because of the surge of new members enrolled in Medicaid after expansion, appointments may have been unavailable and new enrollees could have experienced long waiting times (29, 30).

The trend of decreasing utilization of services over the time span of Medicaid expansion (monthly change in average number of visits per person), for both post-Medicaid expansion cohorts is consistent with other studies on Medicaid enrollees that examined the trends of expenditure on outpatient services (31, 32). This trend could be indicative of dissipated pent-up demand that was seen right after Medicaid expansion. This finding could also be due to other social, behavioral, and institutional barriers to access such as unawareness of the importance of postpartum care, lack of time, lack of transportation to service providers, difficulty in getting an appointment, and lack of caregiving support (33, 34).

These findings should be interpreted within the study limitations. Inconsistencies in coding for the type and setting of services used could have resulted in lower numbers compared to the actual numbers of services utilized (35). However, Medicaid data is a more reliable source of data for access and utilization of health care compared to self-reported data (35). We did not explore the potential effect of other ACA provisions intended to increase access to private insurance, such as the health insurance mandate, exchange, changes in payment methods to providers, all of which could have contributed to increased duration of enrollment and utilization of services. Furthermore, we utilized only 2 years of post-Medicaid expansion data which is a relatively short duration to capture the full impact of the policy. Future research should examine the long-term impacts of Medicaid expansion on healthcare service utilization during the postpartum. Because our findings reflect one state’s experience with Medicaid expansion, they may not be generalizable to other states that differ in terms of demographics, population health, political climate, and how Medicaid is managed. The results can, however, provide valuable insights for other states with similar characteristics that have not expanded Medicaid. Finally, in this study, identification of the effect of Medicaid expansion came from single interrupted time series (SITS) estimation and our findings remain robust to different time trends (e.g., quadratic and cubic functional forms). Further, we are unaware of any other important policy or system changes that occurred during the study period and therefore might serve as potential threats to the internal validity of our SITS analysis. Nonetheless, our findings should be interpreted with caution that ITS is subject to potential misspecification bias, especially bias from functional form misspecifications in the absence of an appropriate comparison group.

## Conclusion

Medicaid expansion in Oregon was significantly associated with enrollment duration and utilization of outpatient care by low-income birthing individuals during the postpartum period. These improvement could potentially contribute to reducing mortality and morbidity among birthing individuals in the United States (36). In addition to studying the impact of expanding Medicaid coverage on the use of postpartum services, future research is also needed to examine other social, behavioral, and infrastructural barriers that may limit utilization of services for low-income birthing individuals.

## Data availability statement

The data analyzed in this study is subject to the following licenses/restrictions: The datasets presented in this article are not readily available because the current Data Use Agreement between Oregon State University (OSU) and Oregon Health Authority (OHA) does not allow public release of any data files including original data from OHA and analytic data files constructed by OSU. Requests to access these datasets should be directed to marie.harvey@oregonstate.edu.

## Author contributions

SP and SH conceived and designed the manuscript. SP performed statistical analysis in consultation with LB and JY. SP drafted the manuscript. SP, SH, LB, and JY revised the manuscript for important intellectual content. All authors have made substantial contribution to the manuscript, read, and approved the submitted version.

## Conflict of interest

The authors declare that the research was conducted in the absence of any commercial or financial relationships that could be construed as a potential conflict of interest.

## Publisher’s note

All claims expressed in this article are solely those of the authors and do not necessarily represent those of their affiliated organizations, or those of the publisher, the editors and the reviewers. Any product that may be evaluated in this article, or claim that may be made by its manufacturer, is not guaranteed or endorsed by the publisher.
