# Supplementary material for: Postpartum Medicaid coverage and outpatient care utilization among low-income birthing individuals in Oregon: impact of Medicaid expansion
Source: Front Public Health. 2023 Jul 4;11:1025399. doi: 10.3389/fpubh.2023.1025399 (PMC10352675; doi:10.3389/fpubh.2023.1025399)
Supplement: Supplementary file 1 [file Data_Sheet_1.pdf]

APPENDIX

FIGURES

Figure 1. Inclusion criteria for the study sample of Oregon women who had Medicaid financed deliveries between 2011 and 2015

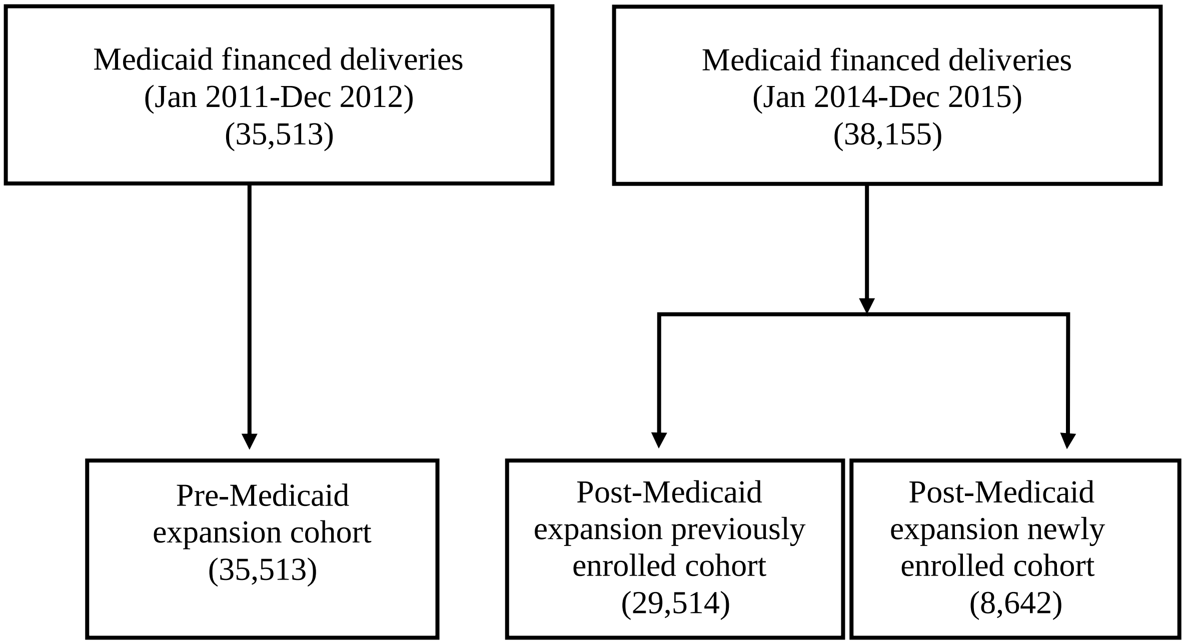

Figure 2: Trends in duration of enrollment during the 12-month postpartum period by cohort for Medicaid-enrolled Oregon women in 2011-2015

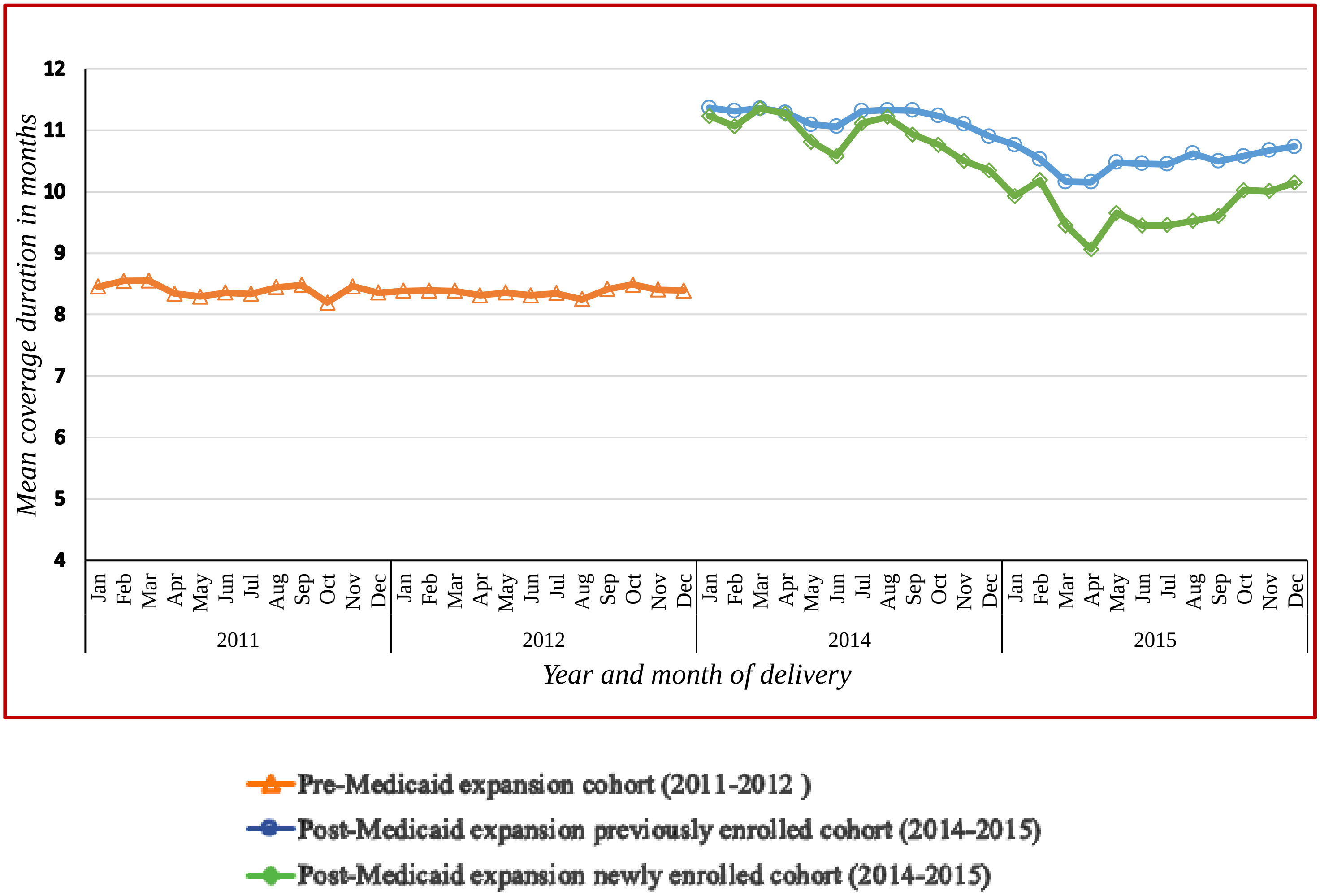

Figure 3: Average number of outpatient visits during the postpartum periods by cohort for Medicaid-enrolled Oregon women in 2011-2015

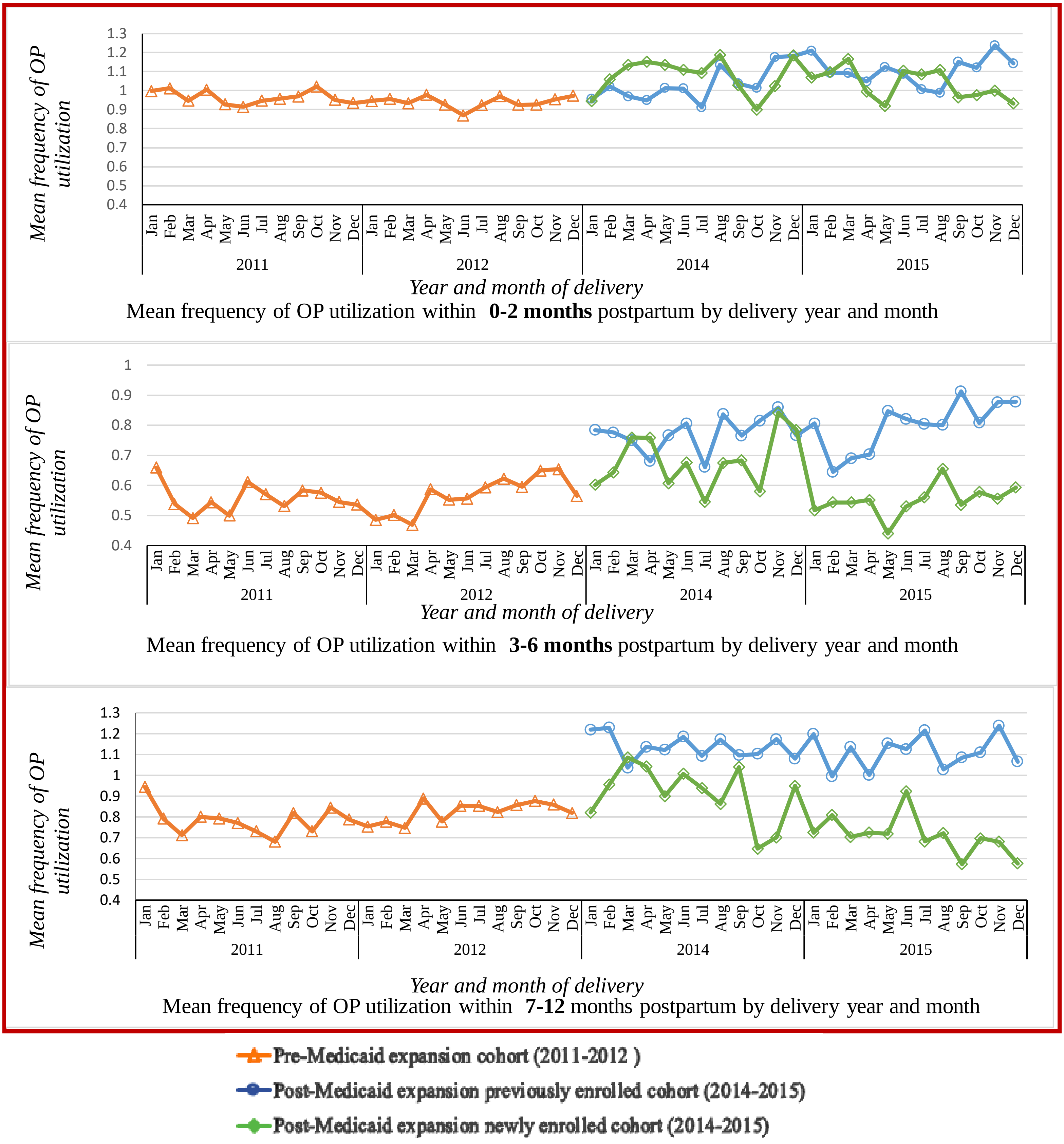

CPT codes used to identify outpatient visits

59400, 59510, 59610, 59618, 90791, 90792, 90832, 90834, 90837, 92625, 96116, 96118, 96150, 96151, 97165 – 97167, 98960-98962, 99078, 99201-99205, 99211-99215, 99241-99245, 99341-99350, 99381-99387, 99391-99397, 99401-99404, 99411, 99412, 99483, 99510, G0101, G0155, G0176, G0177, G0402, G0409, G0438, G0439, G0444, G0502 - G0505, G0507, G8510, H0002, H0004, H0031, H0034, H0036 - H0040, H2000, H2011, H2013 - H2020, M0064, T1015.

Table 5: Unadjusted regression models for mean coverage duration and outpatient care utilization

Outcome	Post-Medicaid expansion previously enrolled cohort	Post-Medicaid expansion newly enrolled cohort
	**Coefficient[1] (CI)**	**Coefficient[1] (CI)**
Number of months covered after delivery	2.51***(2.46 - 2.57)	1.04 (1.00 - 1.08)
**Outpatient care utilization**		
**0-2 months**		
Average number of visits per person during post-Medicaid expansion period (overall)	0.12***(0.10 – 0.13)	0.11***(0.08 – 0.14)
**3-6 months**		
Average number of visits per person during post-Medicaid expansion period (overall)	0.34*** (0.30 – 0.37)	0.09*** (0.04 – 0.14)
**7-12 months**		
Average number of visits per person during post-Medicaid expansion period (overall)	0.34*** (0.30 – 0.37)	0.04*** (–0.01 – 0.10)

*** p<0.001 ** p<0.01 * p<0.05

[1]*Reference group was Pre-Medicaid expansion cohort*

Table 6: Full result from negative binomial regression model on number of outpatient care visits by during 0-2 months postpartum among post Medicaid expansion previously enrolled cohort

Birth cohort & covariates	No. of months covered after delivery	
	Coefficient[1]	95% CI
*Time trend*Post-Medicaid expansion newly covered cohort*	0.006	0.003 - 0.008
Post	0.03	–0.01 - 0.07
Individuals's age	–0.002	–0.005 - – 0.000
RUCA (Reference: Urban)		
Large rural	–0.03*	–0.07 - 0.00
Small & Isolated rural	0.02	–0.04 - 0.08
Race/ Ethnicity (Reference: White)		
NH Black	–0.25***	–0.31 - –0.17
NH Asian	–0.20***	–0.27 - –0.14
NH AIAN	0.24***	0.15 - 0.32
NH PI	–0.56***	–0.68 - –0.43
NH Others	–0.17*	–0.30 - –0.03
Hispanic	–0.57***	–0.60 - –0.54
Individuals' Education (Reference: Less than Highschool)		
Highschool & some college	0.21***	0.18 - 0.23
Bachelor's degree	0.29***	0.25 - 0.33
Graduate degree	0.27***	0.17 - 0.36
Marital status (Reference: Unmarried)		
Married	–0.11***	–0.13 - –0.09
Mode of Delivery (Reference: Spontaneous)		
Assisted vaginal	–0.002	–0.069 - 0.063
Cesarean	–0.05***	–0.08 - –0.02
Morbidity in the birthing individual (Reference: Absent)		
Present	0.05	–0.01 - 0.11
Infant morbidity (Reference: Absent)		
Present	0.16***	0.12 - 0.20
Previous pregnancies (Reference: No previous pregnancies)		
1	0.04**	0.02 - 0.07
2	0.12***	0.07 - 0.16
>=3	0.18***	0.13 - 0.23
Antenatal visits (Reference: Zero antenatal visits)		
>=1	0.02***	0.02 - 0.02

*** p<0.001 ** p<0.01 * p<0.05

Abbreviation: NH, Non-Hispanic
AIAN, American Indian Alaskan Native
PI, Pacific Islander

[1]*Reference group was Pre-Medicaid expansion cohort*

Table 7: Full result from negative binomial regression model on number of outpatient care visits by during 0-2 months postpartum among post Medicaid expansion newly enrolled cohort

Birth cohort & covariates	No. of months covered after delivery	
	Coefficient[1]	95% CI
*Time trend*Post-Medicaid expansion newly covered cohort*	–.003***	–0.007 - 0.002
Post	0.18	–0.005 - 0.07
Individual's age	–0.002	–0.005 – 0. 000
RUCA (Reference: Urban)		
Large rural	–0.04	–0.08 - 0.00
Small & Isolated rural	0.02	–0.05 - 0.08
Race/ Ethnicity (Reference: White)		
NH Black	–0.25***	–0.33 - –0.16
NH Asian	–0.30***	–0.38 - –0.22
NH AIAN	0.23***	0.13 - 0.32
NH PI	–0.65***	–0.79 - –0.49
NH Others	–0.21***	–0.36 - –0.05
Hispanic	–0.66***	–0.70 - –0.62
Individual's Education (Reference: Less than Highschool)		
Highschool & some college	0.26***	0.22 - 0.30
Bachelor's degree	0.30***	0.25 - 0.35
Graduate degree	0.26***	0.14 - 0.37
Marital status (Reference: Unmarried)		
Married	–0.08***	–0.11 - –0.05
Mode of Delivery (Reference: Spontaneous)		
Assisted vaginal	–0.06	–0.13 - 0.02
Cesarean	–0.05*	–0.08 - –0.02
Morbidity in the birthing individual (Reference: Absent)		
Present	0.03	–0.04 - 0.11
Infant morbidity (Reference: Absent)		
Present	0.13***	0.08 - 0.18
Previous pregnancies (Reference: No previous pregnancies)		
1	0.06***	0.03 - 0.10
2	0.16***	0.10 - 0.21
>=3	0.19***	0.12 - 0.25
Antenatal visits (Reference: Zero antenatal visits)		
>=1	0.02***	0.02 - 0.03

*** $p<0.001$ ** $p<0.01$ * $p<0.05$

Abbreviation: NH, Non-Hispanic
AIAN, American Indian Alaskan Native
PI, Pacific Islander

[1]*Reference group was Pre-Medicaid expansion cohort*

Table 8: Full result from negative binomial regression model on number of outpatient care visits by during 3-6 months postpartum among post Medicaid expansion previously enrolled cohort

Birth cohort & covariates	No. of months covered after delivery	
	Coefficient[1]	95% CI
*Time trend*Post-Medicaid expansion newly covered cohort*	0.005	0.001 - 0.009
Post	0.26	0.20 - 0.32
Individual’s age	–0.002	–0.005 - 0.001
RUCA (Reference: Urban)		
Large rural	0.03	–0.03 - 0.08
Small & Isolated rural	–0.02	–0.10 - 0.07
Race/ Ethnicity (Reference: White)		
NH Black	–0.32***	–0.42 - –0.21
NH Asian	–0.44***	–0.58 - –0.29
NH AIAN	0.23***	0.11 - 0.34
NH PI	–1.15***	–1.37 - –0.92
NH Others	–0.30	–0.61 - 0.02
Hispanic	–0.91***	–0.95 - –0.86
Individual’s Education (Reference: Less than Highschool)		
Highschool & some college	0.20***	0.15 - 0.24
Bachelor’s degree	0.14***	0.07 - 0.22
Graduate degree	–0.18	–0.37 - 0.02
Marital status (Reference: Unmarried)		
Married	–0.32***	–0.36 - –0.29
Mode of Delivery (Reference: Spontaneous)		
Assisted vaginal	0.07	–0.05 - 0.18
Cesarean	–0.05***	0.06 - 0.14
Morbidity in the birthing individual (Reference: Absent)		
Present	0.01	–0.10 - 0.12
Infant morbidity (Reference: Absent)		
Present	0.28***	0.22 - 0.34
Previous pregnancies (Reference: No previous pregnancies)		
1	0.16***	0.11 - 0.21
2	0.30***	0.23 - 0.36
>=3	0.43***	0.35 - 0.50
Antenatal visits (Reference: Zero antenatal visits)		
>=1	0.02***	0.01 - 0.02

*** p<0.001 ** p<0.01 * p<0.05

Abbreviation: NH, Non-Hispanic
AIAN, American Indian Alaskan Native
PI, Pacific Islander

[1]*Reference group was Pre-Medicaid expansion cohort*

Table 9: Full result from negative binomial regression model on number of outpatient care visits by during 3-6 months postpartum among post Medicaid expansion newly enrolled cohort

Birth cohort & covariates	No. of months covered after delivery	
	Coefficient[1]	95% CI
*Time trend*Post-Medicaid expansion newly covered cohort*	–.007	–0.02 - 0.00
Post	0.21***	0.11 - 0.30
Individual's age	–0.003	–0.008 – 0. 001
RUCA (Reference: Urban)		
Large rural	0.05	–0.02 - 0.13
Small & Isolated rural	–0.07	–0.18 - 0.05
Race/ Ethnicity (Reference: White)		
NH Black	–0.35***	–0.49 - –0.22
NH Asian	–0.55***	–0.73 - –0.38
NH AIAN	0.23**	0.09 - 0.39
NH PI	–1.24***	–1.55 - –0.93
NH Others	–0.32	–0.67 - 0.02
Hispanic	–1.01***	–1.07 - –0.94
Individual's Education (Reference: Less than Highschool)		
Highschool & some college	0.20***	0.13 - 0.25
Bachelor's degree	0.09	0.003 - 0.18
Graduate degree	–0.20	–0.42 - 0.02
Marital status (Reference: Unmarried)		
Married	–0.35***	–0.40 - –0.30
Mode of Delivery (Reference: Spontaneous)		
Assisted vaginal	–0.002	–0.14 - 0.14
Cesarean	0.10***	0.05 - 0.16
Morbidity in the birthing individual (Reference: Absent)		
Present	0.004	–0.13 - 0.14
Infant morbidity (Reference: Absent)		
Present	0.28***	0.20 - 0.36
Previous pregnancies (Reference: No previous pregnancies)		
1	0.17***	0.11 - 0.23
2	0.36***	0.26 - 0.44
>=3	0.50***	0.40 - 0.61
Antenatal visits (Reference: Zero antenatal visits)		
>=1	0.02***	0.01 - 0.02

*** p<0.001   ** p<0.01   * p<0.05

Abbreviation: NH, Non-Hispanic
AIAN, American Indian Alaskan Native
PI, Pacific Islander

[1]*Reference group was Pre-Medicaid expansion cohort*

Table 10: Full result from negative binomial regression model on number of outpatient care visits by during 7-12 months postpartum among post Medicaid expansion previously enrolled cohort

Birth cohort & covariates	No. of months covered after delivery	
	Coefficient[1]	95% CI
*Time trend*Post-Medicaid expansion newly covered cohort*	–0.002	0.006 - 0.001
Post	0.37	0.31 - 0.43
Individual's age	0.001	–0.003 - 0.004
RUCA (Reference: Urban)		
Large rural	–0.02	–0.07 - 0.03
Small & Isolated rural	–0.03	–0.11 - 0.05
Race/ Ethnicity (Reference: White)		
NH Black	–0.29***	–0.38 - –0.20
NH Asian	–0.46***	–0.61 - –0.31
NH AIAN	0.22***	0.11 - 0.34
NH PI	–0.74***	–0.93 - –0.55
NH Others	–0.23	–0.54 - 0.08
Hispanic	–0.89***	–0.94 - –0.84
Individual's Education (Reference: Less than Highschool)		
Highschool & some college	0.20***	0.15 - 0.24
Bachelor's degree	0.13**	0.05 - 0.20
Graduate degree	–0.17	–0.40 - 0.06
Marital status (Reference: Unmarried)		
Married	–0.32***	–0.36 - –0.28
Mode of Delivery (Reference: Spontaneous)		
Assisted vaginal	0.002	–0.11 - 0.12
Cesarean	0.10***	0.06 - 0.15
Morbidity in the birthing individual (Reference: Absent)		
Present	0.01	–0.09 - 0.12
Infant morbidity (Reference: Absent)		
Present	0.28***	0.22 - 0.34
Previous pregnancies (Reference: No previous pregnancies)		
1	0.16***	0.11 - 0.20
2	0.30***	0.24 - 0.37
>=3	0.50***	0.43 - 0.57
Antenatal visits (Reference: Zero antenatal visits)		
>=1	0.02***	0.02 - 0.02

*** p<0.001 ** p<0.01 * p<0.05

Abbreviation: NH, Non-Hispanic
AIAN, American Indian Alaskan Native
PI, Pacific Islander

[1]*Reference group was Pre-Medicaid expansion cohort*

Table 11: Full result from negative binomial regression model on number of outpatient care visits by during 7-12 months postpartum among post Medicaid expansion newly enrolled cohort

Birth cohort & covariates	No. of months covered after delivery	
	Coefficient[1]	95% CI
*Time trend*post-Medicaid expansion newly covered cohort*	–0.02***	–0.03 - 0.01
Post	0.30***	0.20 - 0.39
Individual's age	0.00	–0.00 – 0. 00
RUCA (Reference: Urban)		
Large rural	0.02	–0.05 - 0.10
Small & Isolated rural	–0.07	–0.19 - 0.06
Race/ Ethnicity (Reference: White)		
NH Black	–0.22***	–0.49 - –0.22
NH Asian	–0.58***	–0.73 - –0.38
NH AIAN	0.17*	0.09 - 0.39
NH PI	–0.78***	–1.02 - –0.53
NH Others	–0.32	–0.66 - 0.05
Hispanic	–1.01***	–1.09 - –0.95
Individual's Education (Reference: Less than Highschool)		
Highschool & some college	0.20***	0.14 - 0.26
Bachelor's degree	0.07	–0.03 - 0.16
Graduate degree	–0.12	–0.39 - 0.15
Marital status (Reference: Unmarried)		
Married	–0.37***	–0.42 - –0.32
Mode of Delivery (Reference: Spontaneous)		
Assisted vaginal	–0.05	–0.20 - 0.08
Cesarean	0.10***	0.05 - 0.16
Morbidity in the birthing individual (Reference: Absent)		
Present	0.00	–0.13 - 0.13
Infant morbidity (Reference: Absent)		
Present	0.30***	0.22 - 0.37
Previous pregnancies (Reference: No previous pregnancies)		
1	0.18***	0.13 - 0.24
2	0.37***	0.28 - 0.45
>=3	0.59***	0.49 - 0.68
Antenatal visits (Reference: Zero antenatal visits)		
>=1	0.02***	0.02 - 0.03

*** p<0.001 ** p<0.01 * p<0.05

Abbreviation: NH, Non-Hispanic
AIAN, American Indian Alaskan Native
PI, Pacific Islander

[1]*Reference group was Pre-Medicaid expansion cohort*

Analytical models:

Model for change in duration of postpartum coverage:

$$Y_{it} = g(\beta_0 + \beta_1 postMedicaid\ expansion\ previously\ enrolled\ cohort_i + \beta_2 postMedicaid\ expansion\ newly\ enrolled\ cohort_i + \beta_3 timetrend_t + x'_i\beta)$$

Where Y is the number of postpartum coverage months for delivery i at time t, and **g** is the linear index function. The vector x' includes all covariates listed in Table 2.

Model for change in utilization of outpatient care for post-Medicaid expansion previously enrolled cohort:

$$Y_{it} = \Gamma(\beta_0 + \beta_1 post\text{–}Medicaid\ expansion\ delivery_i + \beta_2 timetrend_t * postMedicaid\ expansion\ previously\ enrolled\ cohort_i + x'_i\beta)$$

Where Y is the number of outpatient visits during the postpartum period for delivery i at time t, and Γ is the exponential function. The vector x' includes all covariates listed in Table 2.

Model for change in utilization of outpatient care for post-Medicaid expansion newly enrolled cohort:

$$Y_{it} = \Gamma(\beta_0 + \beta_1 post\text{–}Medicaid\ expansion\ delivery_i + \beta_2 timetrend_t * postMedicaid\ expansion\ newly\ enrolled\ cohort_i + x'_i\beta)$$

Where Y is the number of outpatient visits during the postpartum period for delivery i at time t, and Γ is the exponential function. The vector x' includes all covariates listed in Table 2.
